# Touch Interacts with Vision during Binocular Rivalry with a Tight Orientation Tuning

**DOI:** 10.1371/journal.pone.0058754

**Published:** 2013-03-05

**Authors:** Claudia Lunghi, David Alais

**Affiliations:** 1 Department of Neuroscience, Università Degli Studi di Firenze, Firenze, Italy; 2 Institute of Neuroscience, Consiglio Nazionale delle Ricerche, Pisa, Italy; 3 School of Psychology, University of Sydney, Sydney, New South Wales, Australia; Radboud University Nijmegen, The Netherlands

## Abstract

Multisensory integration is a common feature of the mammalian brain that allows it to deal more efficiently with the ambiguity of sensory input by combining complementary signals from several sensory sources. Growing evidence suggests that multisensory interactions can occur as early as primary sensory cortices. Here we present incompatible visual signals (orthogonal gratings) to each eye to create visual competition between monocular inputs in primary visual cortex where binocular combination would normally take place. The incompatibility prevents binocular fusion and triggers an ambiguous perceptual response in which the two images are perceived one at a time in an irregular alternation. One key function of multisensory integration is to minimize perceptual ambiguity by exploiting cross-sensory congruence. We show that a haptic signal matching one of the visual alternatives helps disambiguate visual perception during binocular rivalry by both prolonging the dominance period of the congruent visual stimulus and by shortening its suppression period. Importantly, this interaction is strictly tuned for orientation, with a mismatch as small as 7.5° between visual and haptic orientations sufficient to annul the interaction. These results indicate important conclusions: first, that vision and touch interact at early levels of visual processing where interocular conflicts are first detected and orientation tunings are narrow, and second, that haptic input can influence visual signals outside of visual awareness, bringing a stimulus made invisible by binocular rivalry suppression back to awareness sooner than would occur without congruent haptic input.

## Introduction

When incompatible images are presented to corresponding retinal regions, interocular differences prevent the brain from achieving binocular fusion and normal binocular vision is not possible. Instead, the conflicting images trigger a continual struggle for visual awareness in which only one image is perceived at a time and the other is suppressed from awareness. These perceptual alternations, a form of bistable perception known as binocular rivalry [1], [2], will continue irregularly each second or two for as long as the conflicting stimuli are present. Because of the peculiar dissociation between a continuous physical stimulation but an alternating visual perception, binocular rivalry has been used as a tool to investigate the neural correlates of visual awareness and the resolution of perceptual ambiguity [3], [4], [5].

The resolution of perceptual ambiguity is thought to be one of the main functions of cross-modal interactions [5], [6]. In the context of binocular rivalry or other bistable visual stimuli, a number of recent studies have shown that non-visual modalities can influence the dynamics of visual alternations, including sound [7], [8], [9], [10], [11], [12], touch [13], [14], [15], [16], [17] and olfaction [18]. Most cross-modal studies that have used bistable visual perception could be interpreted in principle as a shift of attention provoked by an unambiguous signal in another sensory modality. Most studies, in fact, have shown that cross-sensory stimulation can extend the dominance duration of the congruent rivalry stimulus. This effect is similar of that of attention on binocular rivalry [19], [20] in which attentional selection of one image tends to prolong dominance of the attended stimulus but generally does not shorten the suppression period ([19], [21]; but see also [22]). In line with this hypothesis, van Ee et al [12] have demonstrated that cross-modal stimulation (either auditory or tactile) can enhance attentional control over binocular rivalry even though, in their paradigm, it was ineffective automatically and required a conscious attentional act to be effective.

Recently, Lunghi et al [14] showed that touching an oriented haptic grating congruent with one or the other rivaling images extended the dominance duration of the parallel visual stimulus. In addition, and unlike the effects of attention on rivalry, this study demonstrated that the haptic signal shortened the period of suppression of the congruent visual signal and restored it more quickly to consciousness than in a visual-only condition. This implies that the haptic signal interacts with the congruent visual signal outside of awareness (i.e., while it is suppressed). They also showed that the influence of haptic stimulation on the dynamics of binocular rivalry was tuned for spatial frequency in that visual and haptic gratings with the same spatial frequency produced an effect whereas a difference of one octave eliminated it. From these observations (haptic interaction during suppression and spatial frequency tuning), and given that neural signals associated with the suppressed image are not traceable outside of V1–V2 [23], [24] and that neurons with narrow spatial tunings are only found in primary visual cortex [25], their data were interpreted as evidence of an early, compulsory interaction between vision and touch. Another study recently drew the same conclusion for audio-visual frequency interactions [26].

The conclusion that vision and touch interact early in visual processing squares with other recent evidence. Multisensory convergence in primary visual cortex has been found in the macaque brain [27], and recently the primary visual cortex of rodents has been shown to respond to haptic exploration of novel objects [28], its response correlating with the animal’s performance in a tactile aperture discrimination task. In humans, V1 activity (BOLD) has been found in response to tactile stimulation [29], moreover, the primary visual cortex is recruited for tactile processing both in blind patients [30] and in blindfolded normal-sighted adults [31]. The early visual-touch interaction suggested by Lunghi et al [14] fits with recent evidence of multisensory convergence and with a proposal that the whole brain is fundamentally multisensory, including areas traditionally thought of as primary sensory cortices [32].

In the current paper we further investigate the hypothesized early interaction between vision and touch during binocular rivalry by testing whether it is specific for another basic property of the primary visual cortex, fine *orientation selectivity* [33], a point that was not addressed in the paper by Lunghi et al [14]. Using the same paradigm as Lunghi et al [14], we confirmed that the effect of congruent touch in binocular rivalry acts in two ways, both extending the dominance duration of the visible stimulus and reducing the time that the invisible stimulus is suppressed. Importantly, by varying the relative orientation of the visual and haptic gratings, we show that this effect of touch on vision is strictly orientation tuned, reinforcing the hypothesis that the interaction occurs at the earliest stages of visual analysis, probably V1. Finally, by showing that observers were unaware of the mismatched orientation between the visual and the haptic stimuli we also extended the results obtained by Lunghi et al: observers’ inefficiency in consciously perceiving the visuo-haptic mismatch in orientation, in fact, reduces the possibility that the fine orientation tuning that we found could be attributable either to categorical perception or to response bias.

## Materials and Methods

### Subjects

Eight subjects (four males, average age 28.3±7.3 years), including the authors, participated in the main experiment (subject JT took part in only five conditions) and five subjects from this group also participated in the orientation discrimination experiment; all had normal or corrected-to-normal vision and no strong eye preference.

### Ethics Statement

Participants gave written informed consent. The experimental procedure conformed to the declaration of Helsinki and was approved by the local ethics committee (Human Research Ethics Committee (HREC) Low Risk Executive Committee, University of Sydney, Protocol No. 14893).

### Apparatus and Stimuli

**Figure 1:**
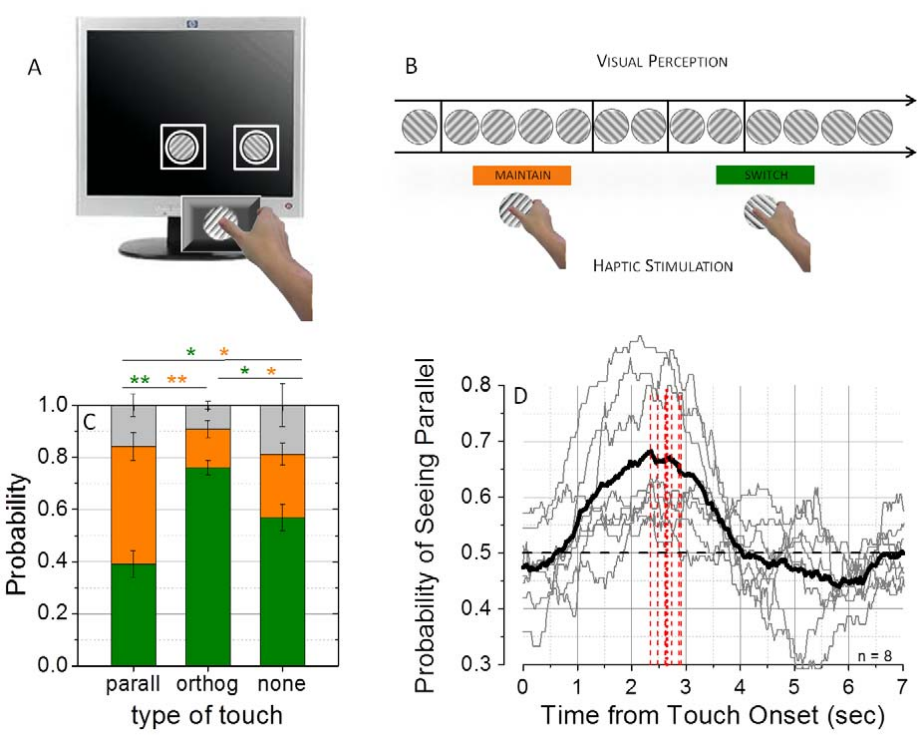
Experimental design and results for matched visuo-haptic orientations. Panel A shows a diagram of the experimental setup: orthogonal gratings (±45° relative to vertical) were presented separately to the eyes through a mirror stereoscope in order to produce binocular rivalry, the haptic stimulus (engraved grating) was placed underneath the monitor and laid on the same plane as the visual stimuli. During binocular rivalry, at random intervals, observers were asked to explore the haptic stimulus with the right thumb. At each touch period visuo-haptic stimulation could randomly be either parallel or orthogonal to the visual stimulus dominating observers’ perception (Panel B). During a touch period observers could either maintain the same image dominant, or switch towards the previously suppressed one. Panel C and D show the results obtained when the haptic stimulus matched the orientation of the visual gratings. The probability of switching, maintaining or switching more than once conditioned to the type of visuo-haptic stimulation (parallel, orthogonal or no-touch) is plotted in Panel C. When visuo-haptic stimulation was orthogonal, the probability of switching (green bars) significantly increased compared both to parallel stimulation (+37%) and no touch periods(+19%). Conversely, the probability of maintaining (orange bars) significantly increased for parallel visuo-haptic stimulation, both compared to orthogonal stimulation (+30%) and to no-touch periods (paired samples t-test, two tailed, α  =  0.05, N = 8, **p≤0.01, *p≤0.05 ). The time-course of the effect of touch on binocular rivalry is reported in Panel D, where the average instantaneous probability of perceiving the visual stimulus parallel to the haptic orientation (black line) is plotted as a function of time elapsed from the onset of touch (the grey thin lines represents individual observers traces). The probability trace is significantly biased in favour of the haptic orientation 1.05 seconds after the onset of touch (t-test, two tailed, α  =  0.05, N = 8), the probability peaks 2.35 seconds after the onset of touch and slowly decays to chance level after the offset of touch (the thick red line represents the average touch duration, while the thin lines the individual observers’ ones).

The experiment took place in a dark, quiet room. Visual stimuli were created in MATLAB using PsychToolbox [34], and displayed on a 17-inch LCD monitor (Hp 1702), driven at a resolution of 1280×1024 pixels with 60 Hz refresh rate. Observers viewed the visual stimuli through a mirror stereoscope placed 40 cm from the monitor. In the main experiment responses were recorded through the computer keyboard, in the cross-modal orientation discrimination responses were also recorded through a pedal switch. Visual stimuli were two oblique orthogonal achromatic gratings (orientation: ±45°, size: 2.5 cm, spatial frequency 2 cyc/cm, contrast 20%, mean luminance 48 cd/m^2^), surrounded by a white smoothed circle included in a white squared frame (size 3.6 cm) to facilitate binocular fusion, presented on a black uniform background (luminance 0.28 cd/m^2^) in central vision. The haptic stimulus was a sinusoidal grating (size: 3 cm, spatial frequency 2 cyc/cm) created with a 3D printer (a diagram of the experimental setup is reported in Figure 1A). Participants could not see their hand or the haptic stimulus during the experiment. The haptic grating was attached to a shaft and its orientation could be precisely varied by the experimenter using a calibrated switch.

### Task and Procedure

#### Binocular Rivalry

The dynamics of binocular rivalry can be influenced by haptic signals, as previously documented [14]. Here we investigate the orientation tuning of this effect of touch on vision during binocular rivalry. Using the same experimental paradigm as Lunghi et al [14], we varied the orientation of the haptic stimuli in different experimental blocks, introducing a mismatch in orientation with the visual stimuli ranging from ±7.5° to ±30°.

Each observer participated in five 180-sec experimental sessions x seven experimental conditions for a total time of 105 minutes, over different days. Seven different haptic conditions in which a mismatch in orientation between the visual and haptic stimuli was introduced were tested in separate blocks. Within every block two orthogonal haptic orientations were tested, one clockwise, the other counterclockwise relative to vertical. The mismatch in orientation was defined as the difference between the orientation of the visual gratings (±45°) and the haptic gratings whose orientation could be: -30°, -15°, -7.5°, 0°, +7.5°, +15°, or +30° relative to the orientation of the visual. The order of the different conditions was randomized for every observer. The two haptic gratings in a block were always orthogonal to each other. Within each block, this grating pair was rotated by a fixed amount relative to the visual gratings, so that one haptic grating was offset by that amount relative to one visual grating and the other haptic grating was offset by the same amount relative to the other visual grating.

Observers were given time to adjust the stereoscope in order to achieve perfect binocular fusion at the beginning of every experimental block when only the square frames were presented. When ready, observers pressed a key and after an acoustic signal (beep) the visual stimuli appeared. Participants were instructed to report their rivalry fluctuations by indicating continuously which visual orientation (clockwise or counterclockwise) they perceived. They did so by pressing one of two keys on the computer keyboard. With the small stimuli we used, the rivalry percepts in our experiment were generally coherent and unitary, with mixed perception occurring only briefly at the time of percept transitions. At the end of each experimental session the orientation of the rival stimuli were swapped between the eyes to counterbalance any eye dominance effects.

During the 3-minute trials, at approximately regular intervals, observers were asked to explore the haptic stimulus with their right thumb by performing a constant translational movement, until the experimenter gave a stop signal (average touch period: 2.65±0.18 s). The experimenter manipulated the orientation of the haptic stimulus between each touch period by alternating between orthogonal orientations (clockwise or counterclockwise) according to a pre-computed random sequence so that the haptic orientation was unpredictable. A cartoon of the experimental paradigm is reported in Figure 1B. Touch periods were brief to avoid haptic adaptation and maintain the haptic stimulation salient. Touch periods were compared to no-touch control periods. Since the effect of haptic stimulation takes more than 1 second to recover after the offset of touch, we defined the no-touch control periods as starting 1.8 seconds after the offset of touch and matched their duration to that of the touch periods (i.e., 2.65 s).

#### Cross-modal orientation discrimination

In order to evaluate if observers were aware of the orientation mismatch between the visual and the haptic stimuli we ran a cross-modal orientation discrimination experiment. During simulated binocular rivalry, we varied the orientation difference between the visual and the haptic stimuli and asked observers to judge whether the visual and the haptic stimuli had the same orientation or not.

As in the rivalry experiment, observers in the orientation discrimination experiment were given time to adjust the stereoscope in order to achieve perfect binocular fusion at the beginning of every experimental block. When ready, observers initiated the trial sequence with a key-press and the visual stimuli appeared. Binocular rivalry was simulated by binocularly presenting iso-oriented gratings (either +45° or -45° relative to vertical) for durations randomly varying between 2 and 3.5 seconds. To simulate the brief patchwork of gratings often perceived during dominance transitions both orthogonally oriented gratings (+45° and -45° [26]) were presented for a short random duration of 0.5-0.7 s. All transitions from one grating to patchwork to the other grating were temporally smoothed and observers were not told that it was a rivalry simulation and in debriefing sessions none of them reported noticing it was a rivalry mimic condition. Observers tracked their visual perception by continuous key-press as in the first experiment. At approximately regular intervals observers were asked to explore with their right thumb the haptic stimulus that in a given each touch period could have either the same orientation as the visual stimuli (+45° or -45° [26]) or could be mismatched by -7.5°, -15°, +7.5° or +15° relative to the visual stimulus. All clockwise and all counterclockwise haptic orientations were tested in separate blocks. During the touch period (mean touch duration 2.1±1.1 s), observers were required to indicate whether the haptic stimulus was further clockwise or further counterclockwise relative to the visual stimulus, and they did so by pressing the appropriate pedal on a two-pedal floor switch. Only responses registered during the touch period were considered for analysis, meaning in effect that observers had to make their response within about 2 seconds of commencing their touch exploration. In order to validly compare discrimination of the visual-haptic orientation difference across conditions, we considered for analyses only haptic orientations that were tilted “further clockwise” than the visual orientation. This was because performance for the 0° visual-haptic difference showed a bias towards clockwise responses which would spuriously exaggerate differences with haptic gratings tilted counterclockwise. Observers’ performance for haptic orientations tilted “further counterclockwise” did not statistically differ from performances for “further clockwise orientations”.

## Results

Observers reported binocular rivalry alternations between orthogonal gratings while periodically exploring an adjacent haptic grating (Figure 1A) which was randomly varied by the experimenter to be either parallel or orthogonal to the visual stimulus dominating the observer’s perception at that time (Figure 1B). During a touch period observers could either maintain the same visual percept or switch perception in favour of the previously suppressed visual stimulus (Figure 1B), or in a minority of cases more than one switch might occur. We therefore computed the probabilities of switching visual percept, of maintaining the same percept, or of switching more than once during the touch period (conditioned to the type of visuo-haptic stimulation: parallel, orthogonal or for a no-touch control period of visual-only stimulation). If haptic stimulation interacts with binocular rivalry dynamics by promoting dominance of the parallel visual stimulus, we would expect the probability of maintaining the same visual percept for the touch period to be higher for parallel visuo-haptic stimulation, or the probability of switching to be higher for orthogonal visuo-haptic stimulation (or both, as in Lunghi et al [14]).

We found that haptic stimulation promoted dominance of the parallel visual percept both by increasing its dominance and by curtailing its suppression (replicating the results reported by Lunghi et al[14]). When the visual and haptic stimuli were parallel, the probability of *maintaining* the same visual percept during the whole touch period increased by 30% relative to orthogonal visuo-haptic stimulation, and by 21% relative to no-touch control periods. Conversely, when the visual and haptic stimuli were orthogonal, the probability of *switching* visual percept increased by 37% relative to parallel visuo-haptic stimulation and by 19% compared to no-touch periods (Figure 1C, statistics are reported in the figure caption). These results demonstrate that touch specifically interacts with vision during binocular rivalry both by maintaining congruency between the visual and haptic stimuli when the haptic stimulus is parallel to the dominant visual stimulus (retarding rivalry alternations to the orthogonal stimulus in the other eye) and by re-establishing dominance of the congruent visual stimulus when it is rendered invisible by binocular rivalry suppression (boosting the suppressed visual stimulus and reverting it to consciousness). We obtained the same results in a control experiment in which we asked observers to track periods of full dominance of one or the other visual stimulus as well as periods of mixed rivalry. In this control experiment, touch periods starting during a period of mixed rivalry (18% on average) were discarded from analysis, so that the visual stimulus parallel to the haptic stimulus was always completely dominant or completely suppressed from observers’ visual awareness. This confirms that the effect of the haptic stimulus curtailing the duration of the suppressed visual stimulus is genuine and not a spurious one due to partial visibility of the dominant grating.

To examine the time-course of the influence of haptic stimulation on the dynamics of binocular rivalry, we computed the instantaneous probability of perceiving the visual grating parallel to the haptic stimulus as a function of time from the onset of a touch period (collapsing parallel and orthogonal visuo-haptic stimulation). If the two rival stimuli are equally likely to be perceived, the probability trace oscillates around chance level. We found that 1 second after the onset of touch the probability trace is significantly biased towards the visual percept parallel to the haptic stimulus (Figure 1D). The effect peaks at 2.35 seconds and slowly decays to chance level at around 4 seconds after the onset of haptic stimulation (1.4 seconds after its offset).

**Figure 2:**
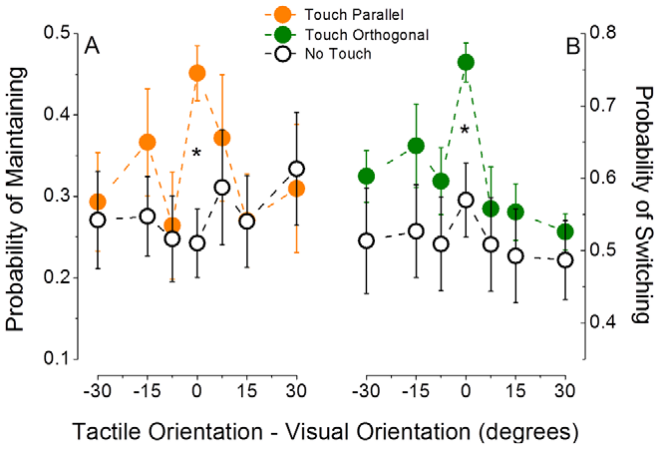
Orientation tuning of the probability of maintaining and switching perception during parallel or orthogonal visuo-haptic stimulation compared to no-touch periods. The probability of maintaining the same visual stimulus during the whole touch period when visuo-haptic stimulation is parallel (Panel A, orange symbols) and the probability of switching perception when visuo-haptic stimulation is orthogonal (Panel B, green symbols), compared to no-touch periods (open symbols) are plotted as a function of the mismatch in orientation between the visual and the haptic gratings (defined as haptic orientation – visual orientation). Both probabilities significantly differ from no-touch periods only when visual and haptic stimuli are perfectly matched in orientation (paired t-test, two-tailed, df = 7, p≤0.05).

The results presented thus far were for haptic gratings that were either parallel or orthogonal to the dominant visual percept and show a significant effect of touch on vision. Is the effect of touch on vision more finely tuned for orientation? Figure 2 plots the probabilities for maintaining visual percept (left-hand panel) and for switching visual percept (right-hand panel) when the haptic stimulus was mismatched in orientation relative to the visual stimuli by ±7.5°, ±15° or ±30°. Unlike the results for a 0° orientation difference reported above, the data in Figure 2 show clearly that neither the probability for maintaining (Figure 2A) nor the probability for switching (Figure 2B) differed from the no-touch control periods when there was a relative orientation difference between the visual and haptic stimuli (statistics reported in the figure caption). While both probabilities peak when visual and haptic stimuli are perfectly matched in orientation (0° difference), the effects of touch at ±7.5°, ±15° and ±30° were not significant for either switching or maintaining. This demonstrates that the interaction between vision and touch during binocular rivalry is strictly orientation tuned.

The fact that the effect of touch on binocular rivalry exhibits an all-or-nothing behavior, being significant for perfect visuo-haptic alignment but not when mismatched by as little as 7.5°, was unexpected. One possibility is that it could be due to categorical perception rather than to sensory tuning *per se*. To investigate this we ran an orientation discrimination experiment in which we asked observers to compare haptic and visual orientations. The central question was whether observers could perceive the orientation difference between a visual grating and a haptic grating with an orientation of +7.5° or -7.5° relative to the visual stimuli orientation. If they were able to discriminate this small difference, it would be consistent with a non-linear decision-making process based on categorical perception. If observers are unable to discriminate the ±7.5° visual- haptic orientation difference, we could exclude the involvement of a categorical decision in mediating the tight orientation tuning of the interaction.

**Figure 3:**
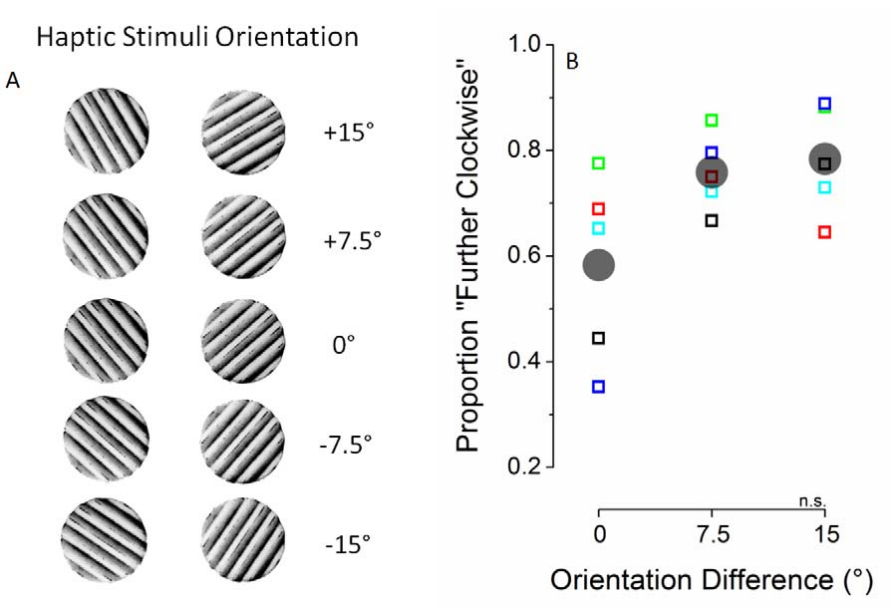
Cross-modal orientation discrimination experiment: haptic stimuli and results. Panel A shows the different haptic orientations used during the experiments. In the main experiment pairs of orthogonal haptic orientations were tested within every experimental block. In the cross-modal orientation discrimination experiment, clockwise and counterclockwise haptic orientations were tested in separate blocks, observers were required to judge to haptic orientation compared to the visual orientation during simulated binocular rivalry. Participants were forced to report whether the haptic stimulus was oriented “further clockwise” or “further counterclockwise” compared to the congruent visual stimulus (either +45° or -45° relative to vertical). Panel B reports the proportion of “further clockwise responses” plotted as a function of the mismatch in orientation between the haptic and the visual stimuli. The small coloured symbols represent individual observers’ performances (every colour representing a different observer), the big transparent grey dots represent the average performance. Even though a trend of improving performance with increasing visuo-haptic orientation difference is present, observers’ performance did not significantly differ from the average performance for visuo-haptic stimuli matched in orientation neither for the 7.5 degrees difference (one sample t-test, df = 4, t = 2.405, p = 0.074), nor for the 15 degrees difference for the 7.5 degrees difference (one sample t-test, df = 4, t = 1.95, p = 0.123), meaning that they were not able to discriminate the difference in orientation between visual and haptic stimuli within 15° difference. A (non significant) bias towards the “further clockwise” response was also observed when visuo-haptic stimuli were matched in orientation.

In the orientation discrimination experiment, similar to the main experiment, the visual stimuli were oriented at ±45° and observers were asked at approximately regular intervals to explore the haptic stimulus with their right thumb. The orientation of the haptic grating was randomly alternated each touch period and could be either clockwise (+45° relative to vertical) with an additional random offset of 0°, ±7.5° or ±15°, or counterclockwise (-45° relative to vertical) with an additional random offset of 0°, ±7.5° or ±15°. A diagram of the haptic orientation pairs is shown in Figure 3A. The visual stimuli alternated in simulated rivalry, as described above, and the observer’s task was to track the visual alternations by continuous keypress, thereby matching the conditions of the discrimination experiment and the original rivalry experiment as closely as possible. Only touch periods in which observers accurately reported the visual orientation were considered for analysis. While tracking their visual perception, observers also judged during touch periods whether the haptic stimulus was tilted clockwise or counterclockwise relative to the visual stimulus (using a two-pedal floor switch). Because the visual stimulus was alternating, in some touch periods the haptic grating was aligned (or nearly so) with the visual grating (0°, ±7.5° or ±15°) while in other touch periods is was about 90° away (90° + 0°, ±7.5° or ±15°), as in the orthogonal visuo-haptic presentation condition during the main binocular rivalry experiment. In either case, the response required was the same (the haptic grating was either clockwise or counterclockwise of the visual grating).

The results of the visuo-haptic orientation discrimination experiment are reported in Figure 3B which plots the proportion of “further clockwise” responses as a function of angular difference. Threshold performance in this orientation discrimination task is 0.50 correct, however for a visual-haptic orientation difference of 0°, there was a bias to respond “clockwise”. That is observers judged the haptic stimulus matching the visual stimulus orientation as being oriented “further clockwise” ∼60% of the time. For this reason, we compared the +7.5° and +15° conditions shown in Figure 3B with average performance in the 0° condition, rather than the unbiased threshold of 50%. Performance for orientation differences of +7.5° and +15° did not significantly exceed performance in the 0° condition, implying that observers were not able to discriminate the orientation difference between visual and haptic stimuli even when they differed by 15°. Importantly, discrimination performance in the “further counterclockwise” conditions (-7.5° and -15°), although not shown, did not differ significantly from the “further clockwise” conditions. These results indicate that participants were not aware of the visuo-haptic mismatch in orientation during the main experiment. That is, even though the effect of touch on rivalry in the main experiment was confined to perfectly aligned visual and haptic stimuli (0° orientation difference), these discrimination data show that observers were not aware of the difference between 0° and 7.5°, or between or 0° and 15°. The lack of awareness of the visuo-haptic orientation difference therefore suggests that the strict orientation tuning found in the main experiment is not likely to be attributable to a categorical decision.

## Discussion

We have shown that exploring a haptic grating while experiencing binocular rivalry between orthogonally oriented visual gratings can substantially influence the alternation dynamics of binocular rivalry. Touching a grating that is congruent with the visual grating being perceived increases the likelihood that it will remain dominant. Conversely, touching a grating that is orthogonal to the visually perceived grating increases the probability that perception will switch to the suppressed grating and therefore align the visual and haptic percepts (Figure 1D). Because the influence of the haptic grating acts not only on the perceptually dominant visual grating but also on the grating rendered invisible by binocular rivalry suppression (in contrast to attentional effects on rivalry, which generally influence only the dominant grating [19], [20]), these results show that the influence of touch on vision during binocular rivalry occurs outside of visual awareness and is therefore an automatic and compulsory interaction.

One interesting aspect of the data is the relatively slow time course of the effect of touch in promoting dominance of the parallel visual stimulus. When we computed the dynamics of the touch effect we found it takes at least 1 second to significantly bias rivalry and takes more than 2 seconds to peak after touch onset (Figure 1C). The slow time course is probably due to the role of adaptation and reciprocal inhibition in determining binocular rivalry dynamics. As was recently shown [35], sensitivity to the two competing visual stimuli slowly changes during a single rivalry phase: initially sensitivity to the dominant stimulus is high and sensitivity to the suppressed stimulus is low. During a rivalry period, the sensitivity difference reduces due to adaptation of the dominant response and a corresponding release from inhibition of the suppressed response, reaching a near-zero difference just prior to a perceptual switch. Clearly, when the sensitivities to the competing stimuli are very similar (near the end of a rivalry period), the potential for haptic input to bias visual competition would be greater. In our paradigm the touch stimulation was delivered at random moments relative to the rivalry process (near the middle of a rivalry phase, on average) and so we would expect the touch effect to increase over time as the current rivalry period extends and the relative strength of the visual stimuli converges. The time needed for touch to reach the peak effect therefore reflects the time-course of visual adaptation, and is not indicative of the time taken for haptic signals to feedback into early visual areas.

One of the striking findings in this study is that the influence of touch on vision in binocular rivalry is orientation tuned, and very narrowly so (Figure 2). Indeed, our data show that the interaction requires matched visuo-haptic orientations, as a mismatch of 7.5° between visual and haptic orientations was sufficient to annul the effect of touch on binocular rivalry. We explored this narrow tuning further in a visuo-haptic orientation discrimination experiment. The results showed that observers were not able to discriminate a 7.5° visuo-haptic orientation difference as being different from a 0° orientation difference (Figure 3). Nonetheless, although they were perceptually indistinguishable in orientation, our orientation tuning experiment established clearly that 7.5° was ineffective at biasing rivalry dynamics (Figure 2). This pattern of orientation tuning shows a selectivity for orientation that is finer than conscious discrimination, suggesting that the unisensory signals do not need to be individually processed before being integrated. This is contrary to what would be expected in an optimal integration framework in which integration is thought not to be mandatory when integrating between the senses and which is thought to occur at a higher-level of processing after unisensory encoding [36].

Fine orientation tuning is a characteristic of both early visual cortex [33] and early somatosensory cortex (see Hsiao et al [37] for a review) and links between visual and somatosensory systems has indeed been demonstrated [38], [39]. In primary visual cortex, single-unit recordings show that cells typically exhibit a sharp orientation tuning with a bandwidth of approximately 15°, a bandwidth consistent with behavioural studies of orientation perception [40]. Narrow orientation tuning of V1 cells has been reported in neurophysiological studies [41], [42]. Given that the interaction between visual and haptic signals during binocular rivalry is tightly tuned for orientation, we conclude it is likely mediated by early visual neurons. This conclusion reinforces the hypothesis first advanced by Lunghi et al [14] that neural signals for touch and vision interact at the earliest stages of visual processing, probably V1. These authors based their conclusion on the fact that the visuo-haptic interaction they observed in binocular rivalry was tightly tuned for spatial frequency. Our finding of a tight orientation-tuned effect of touch on vision perfectly complements their finding and adds converging evidence for an early visuo-haptic interaction.

The orientation tuning of the visuo-haptic interaction that we observed is actually narrower than that shown by visual neurons. One potential explanation of this we considered was that it was a case of ‘categorical perception’, a kind of non-linear perceptual response that can change abruptly around a boundary. Examples of categorical visual perception have been found in face perception [43], familiar objects perception [44] and colour perception [45]. To explain the orientation tuning of our effect, a categorical perceptual response could be envisaged which is thresholded to occur only when the visual and haptic gratings are iso-oriented and otherwise produces a null response. We specifically tested this hypothesis in our discrimination experiment by testing whether subjects were aware of small differences between visual and haptic orientation. The categorical perception hypothesis predicts they would be aware of the small differences because of its all-or-none response around 0°. The fact that participants could not consciously discriminate the visuo-haptic mismatch in orientation within 15° difference (Figure 3) rules out the categorical perception hypothesis and instead suggests that the angular differences tested (0°, ±7.5°, ±15°) fell within a single orientation bandwidth and were therefore difficult to discriminate. The discrimination data also rule out a ‘response bias’ account of the peak interaction at 0° because any tendency to respond in a biased way to 0° would be evident for all orientations since they are perceptually indistinguishable.

Another possible explanation of the narrow visuo-haptic orientation tuning is that it occurs as a result of optimal multisensory integration according to the maximum likelihood estimation (MLE) model. In the MLE model [46], multisensory signals are first encoded by unisensory processes and these estimates are then combined in a weighted linear sum. The weight for each sensory component is proportional to that component’s reliability, given by the inverse of its variance. The model predicts that the combined estimate should have a lower variance than the unisensory estimates because of the following formula: σ^2^_VH_  =  (σ^2^_V_*σ^2^_H_)/(σ^2^_V_+σ^2^_H_), where σ^2^_v_ is the variance of the visual estimate and σ^2^_H_ the variance of the haptic estimate. The maximum improvement (lowering) in variance is by a factor of 2, which occurs when σ^2^_H_  =  σ^2^_V_ [6], [46], [47]. More relevant to orientation bandwidths, this means the visuo-haptic standard deviation (σ_VH_) is reduced by a factor of √2. Could the MLE model therefore explain why the orientation tuning for the visuo-haptic interaction is narrower than is typically found in vision or in haptic perception? We think this is unlikely for several reasons. First, visual and haptic orientation tunings are not equal: the haptic tuning is broader than the visual one [37], [38] and hence the maximum reduction in σ_VH_ of √2 is not expected. Second, even making the assumption of equal bandwidths for visual and haptic orientation perception (which may be warranted because rivalry suppression has been shown to broaden visual orientation tuning [48]), the sharp visuo-haptic orientation tuning we observed is much more than a factor of √2 narrower than the unisensory tunings and so is incompatible with the MLE model.

A possible explanation for the sharp orientation tuning that we found would be to consider the haptic signal acting as a broadly orientation-tuned contrast pedestal. Cross-sensory pedestal effects between vision and touch have been recently found and are thought to reflect an early interaction between the two modalities. Arabzadeh et al [49] demonstrated that a visual flash presented near the fingers during a simple haptic discrimination task was able to reproduce the classical ‘dipper effect’ and improve near-threshold stimulus discriminability, as if the haptic signal had a direct input into the visual mechanism and provided the equivalent of a contrast pedestal. Similarly, in a speed discrimination task, Gori et al [50] showed cross-sensory facilitation between vision and touch that resulted in a two-fold improvement of discrimination thresholds that was specific for matched visuo-haptic motion direction. Together, these two findings suggest that the haptic signal in our experiment, which is likely to be broadly tuned after being remapped into visual coordinates and fed back to early visual cortex, effectively provided a contrast pedestal for vision, thereby improving visual orientation discrimination and producing a very sharp tuning.

Overall, our results add further converging evidence in support of the view that multisensory processing is present even in primary sensory cortices. Support for this view comes from a number of neurophysiological and anatomical studies showing unisensory inputs into other unisensory areas (for review see [32], [51]), as well as from sensory deprivation studies (reviewed in Alais et al [52] ) showing rapid recruitment of primary visual cortex for haptic processing observed in blind patients [30], [53]. Indeed, even temporary loss of sight (e.g., 5 days) is sufficient to induce superior haptic performance in blindfolded individuals [31], [54]. Such rapid recruitment suggests somatosensory connections are not created *ex novo* after sensory deprivation but are already present in primary visual cortex in normal functioning and can be strengthened if needed [55]. In normal subject, these connections are likely to be weak compared to vision and easily masked by strong and reliable visual input. We propose that the inherent signal ambiguity in binocular rivalry, in which two equally salient visual stimuli engage in a struggle for perceptual dominance, allows these relatively weak somatosensory inputs to exert a significant influence on early visual processing.
